# Noise Sensitivities in Dogs: An Exploration of Signs in Dogs with and without Musculoskeletal Pain Using Qualitative Content Analysis

**DOI:** 10.3389/fvets.2018.00017

**Published:** 2018-02-13

**Authors:** Ana Luisa Lopes Fagundes, Lynn Hewison, Kevin J. McPeake, Helen Zulch, Daniel Simon Mills

**Affiliations:** ^1^Centro Universitário de Belo Horizonte, Belo Horizonte, Brazil; ^2^Animal Behaviour Clinic, School of Life Sciences, University of Lincoln, Lincoln, United Kingdom

**Keywords:** anxiety, behaviour, dog, fear, noise sensitivity, pain

## Abstract

Noise sensitivity is a common behaviour problem in dogs. In humans, there is a well-established relationship between painful conditions and the development of fear-related avoidance responses. Whilst it is likely that a relationship exists between noise sensitivity and pain in dogs, this does not appear to have been investigated. The aim of this study was to explore the signs of noise sensitivity in dogs with and without musculoskeletal pain by comparing case histories using qualitative content analysis. Data were extracted from the clinical records of 20 cases of dogs presenting with noise sensitivity seen by clinical animal behaviourists at the University of Lincoln, composed of 2 groups—10 “clinical cases” with pain and 10 “control cases” without pain. Loud noises as a trigger of noise sensitivity were a common theme in both groups but ubiquitous among “clinical cases.” In “clinical cases” (i.e., those where pain was identified), the age of onset of the noise sensitivity was on average nearly 4 years later than “control cases.” In addition, strong themes emerged relating to widespread generalisation to associated environments and avoidance of other dogs in the “clinical cases,” which did not appear in the “control cases.” “Clinical cases” responded well to treatment once the involvement of pain had been identified. Veterinarians and behaviourists should carefully assess dogs with noise sensitivities for pain-related problems especially if presenting with these characteristics.

## Introduction

The term “noise sensitivities” encompasses fear, anxiety, and phobia-based responses to a range of sound-related stimuli, such as loudness, pitch, and suddenness (1). Fearful responses can range from panting, hiding, and escape attempts, to destructiveness and self-injury (1). Nearly half of owners report that their dog shows at least one sign of fear when exposed to loud noises (2), but these cases do not appear to be commonly referred for specialist treatment. In the canine behaviour referral caseload collated from the membership of the Association of Pet Behaviour Counsellors (3), only 8% of dogs were referred for a fear or phobia. This raises welfare concerns as fears/phobias are a welfare problem in their own right, and there are a range of medical considerations associated with the apparent onset of noise sensitivity. These include cognitive dysfunction (4) and less specific medical signs such as gastrointestinal problems (5) and potentially thyroid problems (6, 7) although the causal nature of the association with thyroid function remains a matter of some debate (8). The problem may be iatrogenic, with exogenous corticosteroid administration potentially increasing sensitivity to noise (9, 10). In humans, there is a well-established but complex relationship between painful conditions and the development of fear-related avoidance responses (11, 12), and hypersensitivity to sound has been suggested as an indicator of pain (13). There are sex differences in pain sensitivity after exposure to noise. Rhud and Meagher (14) apparently associated with different sympathetic arousal responses in the two genders (increased arousal and fear in women and more surprise and pain but less arousal in men). Whilst there are grounds for believing that there may be a relationship between noise sensitivity and pain in dogs, this has not been explored empirically in the veterinary literature.

Qualitative research methods, such as content analysis or thematic analysis of case histories, provide a potential way to initially extract important information prior to any quantification of the phenomena identified (15, 16). This approach has been used to examine potential signs of occult musculoskeletal pain in aggressive dogs (17). The aim of this study is to explore the presenting signs related to dogs with noise sensitivity with and without musculoskeletal pain using qualitative content analysis.

## Materials and Methods

The work was approved by the relevant University Ethics Committee. Owners had previously given informed written consent for the material to be used for research. All data were extracted from historic clinical records of cases seen by one of four clinical animal behaviourists from the University of Lincoln’s Animal Behaviour Clinic [DM, HZ, KM, or LH (where veterinary input was provided by DM, HZ, or KM)]. The cases were selected with a presenting complaint related to fear, anxiety, or phobia triggered by noise. Twenty cases were selected: 10 with a focus on musculoskeletal pain (“clinical cases”) and 10 with no identified pain focus (“control cases”). “Control cases” were selected as the next (by date) in the historical records after the “clinical case” that met the matching criteria. Case records included the dog’s medical history, veterinary referral form, owner completed questionnaire (both forms available in supplementary files), clinician’s notes (taken during the consultation and through the follow-up period), and video footage from the consultations.

The Animal Behaviour Clinic works only on referral from other veterinarians who complete a form certifying the health of the patient at referral. During the behaviour consultation, the clinician observes the dog’s gait, posture(s), and mobility (e.g., getting in/out the car, moving around the clinic). Any concerns over musculoskeletal problems are then discussed with the referring veterinarian who carries out further investigations/treatment as required.

Demographic data (breed type, gender, and owner-reported temperament) were collated from the owner-completed questionnaire. Data were extracted in relation to a range of subject areas predefined by DM and HZ, and these are shown in Table 1.

**Table 1:** Predefined subject areas for data extraction in “control cases” and “clinical cases.”

Predefined subject areas for data extraction	Specific information
Details of noise sensitivity	Age at onset Specific trigger(s) Physical signs shown Consistency of signs How the problem had changed from its initial manifestation Any tendency to avoid places associated with the occurrence of noise triggers
Occurrence of other potential behaviour problems	Broadly classified in terms of expressions of positive or negative affect in response to: Familiar and unfamiliar dogs Familiar and unfamiliar people Children Other animals New situations Car travel Separation from the owner
Nature of any musculoskeletal problem	How any diagnoses were established (e.g., physical examination, radiography) Presence of overt signs of pain in specific situations/at specific times (e.g., after exercise)
Treatment-related findings	Treatment(s) prior to the consultation Treatment(s) suggested after the consultation Available outcome information

The case records were subjected to qualitative content analysis (18). Themes were agreed on consensus between DM and HZ based on the relative frequency of the occurrences of the predefined subject areas. A codebook was established by AF and it was reviewed by DM, and a sample was checked for reliability between AF and DM, but not tested statistically.

## Results

Table 2 summarises extracted data in relation to the noise sensitivity and the occurrence of other behaviour problems. The age of dogs at presentation varied widely in both populations (“control cases”: ~2–9 years, average 4 years 7 months; “clinical cases”: 2–11 years, average 5 years 7 months). Both groups were similarly mixed in relation to breed and sex, and there were no entire females in either group. The age of the dogs at onset of the problem was notably higher in the “clinical cases” group. Loud noises (as defined by owners) were a feature of the primary presenting complaint in every subject among “clinical cases” but featured in this way in only 6 of 10 of the control group. Many dogs reacted to multiple triggers in both groups (2.7 triggers/clinical case vs 2.6 triggers/control case).

**Table 2:** Data relating to the noise sensitivities and other related triggers in “control cases” and “clinical cases.”

Feature of noise sensitivities	Control cases (*n* = 10)	Clinical cases (*n* = 10)
Average age at onset of the noise sensitivity (range) (years and months)	2 years 8 months (7 months to 6 years)	6 years 6 months (5 months to 10 years)

Types of noise sensitivity forming primary complaint		
“Loud” noises	6/10	10/10
Fireworks	7/10	7/10

Other triggers related to noise sensitivity response		
Gunshots	4/10	0/10
Aeroplanes	3/10	2/10
Car	2/10	3/10
Motorbike	1/10	0/10
Busy area	1/10	0/10
Young children	1/10	0/10
Wind	1/10	0/10
Dark places	0/10	1/10
Birds	0/10	1/10
Walks	0/10	1/10
Traffic	0/10	1/10

The physical signs of the dogs in response to noises were similar with no strong themes in either group, which could separate “control cases” from “clinical cases.” The most common signs in both groups were shaking/trembling and hiding. However, in 8 of 10 “clinical cases,” the response had generalised substantially (5 to the general location where the sound occurred and 3 to the point of avoiding going out in the car); by contrast, this sort of generalisation was only reported in 2 of 10 of the “control cases” (1 each to the location and car). Nine dogs in the clinical group showed signs of apprehension or avoidance of new situations, and seven dogs in the control group had similar difficulties.

One dog in each group suffered from a separation-related problem, and one dog from each group also exhibited aggression towards unfamiliar dogs. Two dogs within the “control cases” showed overt aggression towards children, and no other subjects showed overt aggressive behaviour towards any other subjects. However, 6 of 10 subjects in each group showed signs of anxiety/avoidance in at least one social situation involving people (familiar or unfamiliar adults or, more commonly, children). The two groups appeared to differ in their general response towards dogs. Eight of the “clinical cases” showed some anxiety/avoidance towards at least some other dogs (this was always towards unfamiliar dogs and in one case was also towards familiar dogs), whereas this only occurred among two of the “control cases” (one dog towards both unfamiliar and familiar dogs and one towards just unfamiliar dogs). Problematic behaviour with car travel (which may or may not have been related to noise) was similar between the two groups, occurring in two “control cases” and three “clinical cases.”

General temperament appeared similar between the two groups: among the “clinical cases,” seven dogs were described as primarily positive and three primarily negative; among the controls, the distribution was 8:2.

In this study, concerns over musculoskeletal problems were confirmed using a range of procedures (some individuals having multiple procedures): four clearly demonstrated pain during physical examination in the clinic, eight were radiographed, and one underwent magnetic resonance imaging. The problems identified or inferred related to the hip (including dysplasia–five subjects), degenerative joint disease of the limbs (four subjects), and focal spondylosis in L2 and L3 (one subject). In six of these cases, the owner commented that the dog seemed to be in some pain and/or the pain worsened after exercise.

Prior to consultation, for noise sensitivity, three “clinical cases” and four controls had received no treatment. Some cases had been given multiple treatments. Of the “clinical cases,” three had been given nutraceuticals (one Zylkene™, one Calmex™, and one an unspecified mixture), one had been given a pheromone product (Adaptil™), and three had been given psychopharmacological medication (clomipramine in one, a combination of selegiline and alprazolam in one, and fluoxetine, amitriptyline, and selegiline in one case at different times prior to referral). Of the “control cases,” five had been given nutraceuticals (four Zylkene™, one Calmex™), two a pheromone product (Adaptil™), and three psychopharmacological medication (one alprazolam, one fluoxetine, and one selegiline).

Following behaviour consultation, all subjects received an individualised behaviour modification plan, which included management strategies, and guidance on counterconditioning and/or desensitisation to certain noise characteristics. Psychopharmacological intervention was recommended in eight of the “clinical cases” and all “control cases.” All “clinical cases” received analgesia and management advice to reduce the risk of exacerbating pain. Responsibility for the choice of analgesia was with the referring clinician, with advice provided by the veterinary behaviourists as required. Non-steroidal anti-inflammatories were used on all “clinical cases.” Adjunctive psychopharmacology was prescribed in eight of these cases (two imepitoin, two fluoxetine and alprazolam, one alprazolam alone, one selegiline, one clomipramine, and one imepitoin). Among the “control cases,” four were prescribed imepitoin and six were prescribed alprazolam used as required (as a sole agent in two, alongside fluoxetine in two, alongside selegiline in two). Selegiline was later replaced by clomipramine in one of these cases, and alprazolam as a sole agent later replaced with imepitoin in another case.

All cases were reported to improve with treatment, except one dog with hip dysplasia, who had been showing signs of noise sensitivity since 5 months of age and whose owner did not elect to administer analgesia for their dog. Eight “clinical cases” and seven “control cases” were considered resolved to the owner’s satisfaction, at the time of the review.

## Discussion

Qualitative studies such as these provide clinically useful insights where current knowledge is lacking, because they provide a level of detail of examination of the issue that is not often apparent in quantitative studies, especially when sample sizes are limited. No attempt should be made to ascribe any statistical significance to these qualitative findings, rather they should be considered the basis for further enquiry and reflection by both practitioners and researchers in the field.

Although the average ages of the dogs at presentation were similar, the average age of onset of the problem was nearly 4 years later in the “clinical cases.” This strong theme of an older age of onset suggests that the pain may develop later in life and that owners seek treatment more readily, perhaps because the appearance of the problem is out of character in the subject. The average age of onset within the control population also suggests that the problem does not simply relate to a lack of habituation as a puppy and that other mechanisms need to be considered for many cases [for a review, see the study by Levine (19)]. Nonetheless, in the absence of a medical problem, aversive experience or change in environment, it should be noted that many behavioural problems typically become evident in early life (20) although a recent exploration into noise reactivity among three dog breeds indicated that social maturity may be an important time of onset of these problems (21). These results are consistent with the suggestion that whenever there is a late age onset to a behaviour problem, medical issues including those related to pain should be carefully evaluated. Veterinarians should ensure that all dogs with behaviour problems and especially those with an unusual pattern of onset receive a thorough physical examination, with a particular focus on orthopaedic issues to detect any focus of pain. This is especially important as “clinical cases” could not be obviously distinguished on the basis of their presenting signs, which were similar to those noted in larger studies [e.g., see the study by Iimura (5)].

Although owners may often miss many signs of fear or anxiety (22), it should be noted that there was a strong theme among the “clinical cases” often generalising their responses to the much wider environment, and this may be a clearer prompt for closer medical evaluation. Avoidance of associated locations needs to be distinguished from the more general apprehension and avoidance of new situations, which was common within both groups (7/10 “control cases” vs 9/10 “clinical cases”). The greater generalisation among cases could be derived from additional classical conditioning of fear-related avoidance associated with pain (11, 12). We hypothesise that noises resulting in a normal startle response may cause muscle tensing that can exacerbate pain. It is worth noting that although sensitivity to loud noises was commonly reported in both groups, this was a feature of all “clinical cases.” It is acknowledged that the definition of “loud noises” was a subjective one based on owner report and not specifically measured. Therefore, it is possible that there may have been differences between owners concerning what “loud” noise constitutes. Auditory stimuli can in themselves be perceived as painful, and dogs may have a lower auditory pain threshold (approximately 95 dB) (23) compared to humans (approximately 130 dB) (24). Therefore, it is likely that if owners perceive the noises to be loud, it would also be loud and potentially painful for their dogs, whether there was a pain focus elsewhere. It is also possible that the presence of a musculoskeletal pain focus and sound sensitivity interact to lower reactivity thresholds to related stimuli.

Comorbidities with other behaviour problems have been described in dogs with noise sensitivities (25). The groups in this study appeared to differ in their general response towards dogs with a strong theme related to problematic behaviour with other dogs among the “clinical cases” that was not apparent among controls. In chronic pain, social play can decrease and aggression towards other dogs can potentially increase (26). A dog may use aggressive behaviour to end an interaction, which is painful (e.g., twisting and turning) or prevent an interaction that they anticipate may be painful (e.g., being approached by a bouncy dog). Both of these responses might contribute to the observation here and deserve further investigation.

The majority of cases in both groups had been administered at least one treatment to assist in the control of the noise sensitivity prior to referral, ranging from pheromones to nutraceuticals and prescription medications. Such a high proportion may suggest that owners are looking for adjuncts to “cure” the problem and only proceed to seek specialist professional advice from a referral clinic when these are ineffective. The chemical interventions described may provide a valuable and necessary adjunct to a successful behaviour modification programmes, but should not be used alone in an attempt to effect a “quick fix” (27). Non-response to anxiolytic medication, while common (27), should also be considered as a prompt for closer medical scrutiny of the case. When considering welfare, it is a cause for concern that noise sensitivities had been present for such a long time before referral occurred.

In cases where pain is suspected, response to trial analgesia is important (26, 28), but failure to respond to one analgesic does not exclude the presence of pain (26). Therefore, it is important to establish the cause and type of pain involved as far as possible to ascertain the most appropriate treatment (which may be a combination of analgesics) for that individual patient (28). Further investigations of the cause of pain (e.g., through diagnostic imaging) is valuable for diagnosing a source of chronic pain, but pain cannot be excluded on the basis of normal radiographs nor can radiographic changes predict the degree of pain (26). A potential limitation of the study is that controls were not subjected to further investigations or trial analgesia. Whilst it is possible that some controls may have had a painful condition, there were no observable signs of a musculoskeletal problem when assessed at the clinic to warrant further investigation.

Whilst it might be diagnostically preferable to assess the response to analgesia before considering the use of anxiolytics, the welfare of the individual patient must be prioritised, and as such, it may be that both psychopharmacology and analgesia are administered concurrently or agents with multiple indications (e.g., amitriptyline) are used to exert multiple effects. It should be noted that once pain is successfully managed, the previously learned associations with noise may persist and require their own targeted behaviour modification programme.

There was a relatively high (for our behaviour clinic) usage of psychopharmacological intervention in the cases reported here (18 of 20). This may reflect a bias towards more severe cases being referred to us, including those who had been non-responsive to other treatments, and should not be considered the norm in general practice.

There was a large proportion of neutered dogs: 9 of 10 of both controls and “clinical cases,” whereas the Pet Animal Welfare Report (29) suggests that nationally only about 71% of dogs are neutered. A study by Spain et al. (30) found that decreasing age at gonadectomy in shelter dogs was associated with an increased risk of developing a noise phobia, but it could not be concluded that neutering is causative of noise phobias.

In conclusion, the most important clinical features identified in cases of noise sensitivity associated with pain were a ubiquitous fearfulness of loud noises, an extensive generalisation of the problem to the wider environment and associated problems with other dogs. Unlike aggressive dogs in pain (17), we did not identify a moody temperament as a feature of these cases, and the age of onset was more typical in dogs older than the norm for this condition. Prognosis seems to be excellent if the case is properly managed following the identification of the role of pain. These results should be considered preliminary until controlled studies with a larger sample size can further explore these identified themes and features.

## Ethics Statement

The work was approved by the relevant University Ethics Committee. Owners had previously given informed written consent for the material to be used for research.

## Author Contributions

The idea for the study was devised by DM and HZ. Cases were all seen by DM, HZ, KM, and LH (with veterinary input from DM, HZ, or KM). AF performed the data extraction from case records. AF, LH, KM, DM, and HZ all contributed ideas to and reviewed the final manuscript.

## Conflict of Interest Statement

The authors declare that the research was conducted in the absence of any commercial or financial relationships that could be construed as a potential conflict of interest.
