# Supplementary material for: Noise Sensitivities in Dogs: An Exploration of Signs in Dogs with and without Musculoskeletal Pain Using Qualitative Content Analysis
Source: Front Vet Sci. 2018 Feb 13;5:17. doi: 10.3389/fvets.2018.00017 (PMC5816950; doi:10.3389/fvets.2018.00017)
Supplement: Supplementary file 1 [file Image_1.PDF]

CERTIFICATION OF APPROVAL

I hereby certify my approval for the client described overleaf to be referred for management of the current behaviour problem to The Animal Behaviour Referral Clinic, Minster House, University of Lincoln, Green Lane, Lincoln, LN6 7DL.

Medical History:

Date of last health check / / Weight Kg

Please indicate if there are current or previous health problems concerning the following and attach appropriate details:

- ☐ Allergic Reactions
- ☐ Gastro-intestinal system
- ☐ Cardiovascular system
- ☐ Respiratory system
- ☐ Endocrinological System
- ☐ Sensory systems
- ☐ Musculo-skeletal system
- ☐ Skin and adnexae
- ☐ Nervous system
- ☐ Urogenital system

Please provide details including date of any blood tests performed including specific organ function tests and assays:

……………………………………………………………………………………..
……………………………………………………………………………………..
……………………………………………………………………………………..

Date and purpose of any general anaesthetics:

……………………………………………………………………………………..

Details of any ongoing medical conditions or treatments

……………………………………………………………………………………..
……………………………………………………………………………………..
……………………………………………………………………………………..

PLEASE ATTACH A FULL COPY OF THIS PATIENT’S MEDICAL HISTORY

Signed: F/MRCVS Date

Please note: we can only accept this referral and form if it is printed and then signed by hand. Electronic signatures are not accepted. Thank you

REQUEST FOR ANIMAL BEHAVIOUR REFERRAL

Case Reference Number

In order to certify your approval for referral and safeguard the well-being of both your clients and their pet, please complete the following form and return it to us with the medical history and appropriate behaviour history form.

Contact Veterinary Surgeon:

Practice Name:

Address:

Post Code

Tel: (inc. STD code)

E-mail address:

Client Name:

Patient name:

Species/Breed:

Age:

Sex inc. neuter status:

Address:

Post Code:

Tel: (inc. STD code)

Brief details of behaviour problem:

Date first noticed:

Has euthanasia been considered?
