# Supplementary material for: Noise Sensitivities in Dogs: An Exploration of Signs in Dogs with and without Musculoskeletal Pain Using Qualitative Content Analysis
Source: Front Vet Sci. 2018 Feb 13;5:17. doi: 10.3389/fvets.2018.00017 (PMC5816950; doi:10.3389/fvets.2018.00017)
Supplement: Supplementary file 2 [file Image_2.PDF]

This form should be completed by the person who spends most time with the dog. Please discuss your responses with the whole family and indicate any areas of disagreement between individuals.
Please include as much information as possible, and please use a separate sheet where necessary.
If possible and safe to do so, please send pictures or video footage relating to the problem.

CANINE BEHAVIOUR CONSULTATION

If you are filling in this form by hand, please sign here:

ABOUT YOU

Surname (Mr/Mrs/Miss/Ms) **First name and initials**

Address

Post code

Tel (day) **Tel (evening)**

Tel (mobile) **Email**

What previous experience do you have with dogs?

Briefly explain the reason for obtaining the animal for whom you would now like help.

Briefly describe your ideal dog here

ABOUT YOUR DOG

Name: **Age:**

Breed (if cross, indicate predominant breed if known otherwise Mongrel or mixed)**:**

Sex (please mark the appropriate boxes):

☐ Male ☐ Female
☐ Neutered ☐ Intact

Vaccination status: **Date last wormed:**

Is your dog insured? ☐ Yes ☐ No **Renewal date:**

Please describe in a few words, the nature of the problem causing concern? (we will ask for more details later). If there is more than one problem please number them in order of importance to each member of the family (no. 1 = most important)

Is your pet currently on any medications or supplements etc. (such as dietary supplements or herbal products)? If yes, please list name and dosage.

Has your pet been on medication for his/her behaviour in the past? If yes, please list name and dosage.

Is your pet on any medication for his/her behaviour now? If yes, please list name and dosage.

Known medical history (especially recurrent problems) – please attach a history from your vet if necessary:

YOUR HOME

Type of home (i.e. flat, etc):

Degree of access by dog:

Please list other household pets, names, species, ages, gender and whether they are spayed or neutered.

Name	Species	Age	Gender / Neutered

Please list names and ages of other family members who live at home and how you would describe the relationship of each family member with your dog:

Name	Age	Relationship with dog

If relevant to the problem, e.g. in the case of guarding behaviour in the home, please provide a floor plan of your home

YOUR DOG'S EARLY HISTORY & DEVELOPMENT

Date of birth: **Age when obtained:**

Source:

Details of early life if known, including litter size, early illness, rehoming etc

How was your dog housetrained? Please include details of the age at which you started training, and how long it took to housetrain your dog?

How did you treat mishaps at this time?

Have you ever attended training classes with this pet? Please give details (when, where, age of pet, what was done)

How did your dog do in class?
☐ Very well ☐ Average ☐ Poor
☐ Was asked to leave ☐ Not sure

Obedience commands known:
☐ Sit ☐ Stay ☐ Down ☐ Give Paw
Other:

Is your dog more obedient in different places or with specific individuals?

If your dog does any tricks, please give some details of them here

If your dog plays games, please give some details of them here

Intelligence: Do you think your dog is good / average / poor?

How do you correct your dog when s/he misbehaves?

How do you reward you dog when s/he has done well?

YOUR DOG'S TEMPERAMENT AND PERSONALITY

How would you describe your dog's personality?

When faced with a new situation how is your dog likely to react if on the lead?

Does the behaviour change if on a lead as opposed to being free?

Does your dog have any known fears or things it dislikes?

Reaction *when out* to: Strangers

**Stranger**	**reaction**
Male person	
Female person	
Children	
Unknown dogs	
Known dogs	

Other animals:

Crowds/busy area:

Please list below and rank the five edible things your dog is most fond of (rank 1st favourite)

1.
2.
3.
4.
5.

Please list below and rank the five toys, items or activities your dog is most fond of (rank 1st favourite)

1.
2.
3.
4.
5.

Has your dog ever bitten or attacked anyone?

YOUR DOG'S ROUTINE:

Describe a typical 24 hours in the life of the pet

Does your pet ever wake you at night? If yes, how often and why?

Do you walk your dog, if so how often and for how long?

Does your dog pull on the lead?

How much time does your dog get to run free each day?

Typically how long is your dog alone without people on any given day?

Who initiates play time, you or the dog?

What types of toys does your pet play with?

What is your dog's favourite pastime?

What is your dog's least favourite pastime?

DIET

Feed type:

Amount:

Time of day:

Fed by whom:

Is your dog protective of food?

Appetite:

How much does your dog drink each day?

Please list any supplements, treats or titbits which your dog receives:

SOCIAL BEHAVIOUR

Has your dog ever shown aggressive behaviour (growling, snarling, snapping or biting) to any member of the household? If so, please give details

Does your dog treat all members of the household in the same? Please give details of any differences.

How does your pet react when visitors come to the house? (barking, door charging, etc)

What is your dog's response to familiar or regular visitors?

Name	Purpose	Time & Days	Dog's Reaction

In general, how does your dog respond to the following types of people visiting your home:

**Visitor**	**Reaction**
Unknown males	
Unknown females	
Unknown children	
Unknown dogs	
Other pets	

Is there any particular group of people that your dog responds to in a specific way? If yes please provide details

Is your pet ever protective over parts of his/her body (especially ears & feet)?

If yes, please give details

Is there any sort of aggression in the following circumstances? (growling, snarling (showing teeth), lunging, nipping, biting). Please fill in the chart on this page

(Y =Yes, N =No, ? = Don't Know. If yes, indicate what level from the list above)

	**Adult Females**	**Adult Males**	**Children**	**Any Specific Individual**
**When handling/grooming**				
**If disturbed when resting**				
**If disciplined**				
**When walking on lead**				
**If trying to take food away**				
**When petting or hugging**				
**If taking any other objects from him/her**				

Does your dog ever show aggression when examined by the vet?

Does your pet ever show inappropriate mounting or other sexual activity?

If so, to whom or what?

THE CURRENT PROBLEM

When did it begin?

How long has it been present?

How old was the pet when it began?

Where does it occur?

With whom?

How frequently does the problem occur? (times per day, per week, per month or per year)

Please describe the first incidence of the behaviour that you can recall, the most recent and one other which you remember, i.e. 3 incidents in total.
First incident:

Most recent incident:

Another incident:

What has been done to correct the problem? Please indicate whether each measure has helped, made no difference or made matters worse

Is the problem getting

☐ Better ☐ Worse ☐ No change

Do you suspect any cause?

Other details of the main complaint:

Any other behavioural problems?

What are the feelings of *each* family member about your pet's present behaviour?

In an ideal world what would you hope to achieve with your dog?

What are the essential changes you need to be able to continue to live with your dog?

Under what circumstances would you consider euthanasia?

Please give any other information you think relevant to the case:

FOR ALL CASES INVOLVING AGGRESSIVE BEHAVIOUR.

Describe the most recent incident and the setting in which it occurred. Try to be as precise as possible, imagine you are drawing a picture. Where was your dog? Where was everyone in relation to your dog? What was everyone doing before the incident? What did your dog do exactly and what did he look like just before and after the event?

Are you aware of a trigger for the behaviour or for similar behaviour in the past?

Were you aware of any warning signs before the incident?

What was your immediate reaction?

What was the reaction of the victim?

How did your dog respond to this reaction?

If there was a bite wound, where was it located on the body and what kind of a wound was it (puncture wound, tear, blue spot, etc)?

Thank you. Please return the completed form to the address on the front page.
